# Supplementary material for: Classification of volcanic ash particles using a convolutional neural network and probability
Source: Sci Rep. 2018 May 25;8:8111. doi: 10.1038/s41598-018-26200-2 (PMC5970178; doi:10.1038/s41598-018-26200-2)
Supplement: Supplementary file 1 — supplementary information [file 41598_2018_26200_MOESM1_ESM.pdf]

Supplementary information

Classification of volcanic ash particles using a convolutional neural network and probability

Daigo Shoji[1*], Rina Noguchi[2], Shizuka Otsuki[3], Hideitsu Hino[4]

1. *Earth-Life Science Institute, Tokyo Institute of Technology, 2-12-1 Ookayama, Meguro-ku, Tokyo*

2. *Volcanic Fluid Research Center, Tokyo Institute of Technology, 2-12-1 Ookayama, Meguro-ku, Tokyo*

3. *Geological Survey of Japan, AIST, 1-1-1 Higashi, Tsukuba, Ibaraki*

4. *The Institute of Statistical Mathematics,10-3 Midori-cho, Tachikawa, Tokyo*

∗ Corresponding author: shoji@elsi.jp

1 Geological background of sampling locations

We collected pyroclast samples from the Izu Peninsula (Japan), Miyakejima Island (Japan), and Myvatn (Iceland). The following sections provide brief geological information for each sampling location.

1.1 Izu Peninsula, Japan

The Funabara, Hachikuboyama, Hachinoyama, Inatori, and Sukumoyama scoria cones on the Izu Peninsula belong to the Higashi-Izu monogenetic volcano group (HIMVG)[1]. These scoria cones are the product of basaltic monogenetic activity in the late Quaternary[1].

Funabara scoria cone The Funabara scoria cone (660 m in bottom diameter, R. Noguchi, unpublished data) is located at the west end of the HIMVG field and on the Neogene basement[2]. The rock type of this scoria cone is olivine basalt[2]. At present, the Funabara scoria cone is being quarried, and has revealed a clear stratigraphic profile (Fig. 1). The edifice consists of red-oxidized scoria layers with reverse grading, and bombs (< 30 cm) are included in some of the layers. At the center of the outcrop, black lava thought to be a conduit was observed. We collected five (limited by the quarrying stages) samples from bottom to top at the outcrop; FN15101201 for the lowest layer and FN15101208 for the uppermost layer (Fig. 1). These samples have sorted grain size distributions, and the modes are -2ϕ for FN15101201 and FN15101208, and -3ϕ for FN15101207 (Supplementary Fig. S1). For FN15101205 and FN15101206, it is difficult to determine the mode of the grain size distribution due to the inclusion of bombs.

Hachikuboyama scoria cone The Hachikuboyama scoria cone (1060 m in bottom diameter, R. Noguchi, unpublished data) is located in the western HIMVG field. The rock type of this scoria cone is pyroxene basalt[3]. The sampling outcrop of Hachikuboyama exhibits chaotic alternate layers of red-oxidized scoria and black volcanic sand (Supplementary Fig.

S2). This structure is thought to be formed by reworking in an unconsolidated state (Y. Suzuki, personal communication). The Hachikuboyama sample (HK15120701) has a sorted grain size distribution with a mode of -2.5ϕ (Supplementary Fig. S1).

Hachinoyama scoria cone The Hachinoyama scoria cone (1340 m in bottom diameter, R. Noguchi, unpublished data) is located in the southern HIMVG field. The rock type of this scoria cone is high-Al basalt[4]. Hachinoyama consists of alternate layers of red-oxidized scoria and black volcanic sand with bombs (maximum 15 cm diameter, Supplementary Fig. S2). The Hachinoyama sample (HN15120701) has a sorted grain size distribution with a mode of -2.0ϕ, and no -4ϕ peak caused by larger size bombs beyond our sieving range (Supplementary Fig. S1).

Inatori scoria cone The Inatori scoria cone (910 m in bottom diameter, R. Noguchi, unpublished data) is located in the southern coastal area of the HIMVG field. The rock type of this scoria cone is tholeiitic basalt[4]. This scoria cone has slightly reversed grading of red-oxidized scoria layers (Supplementary Fig. S2), some of which have fluidal shape bombs (50 cm for the major axes). The Inatori sample (IT15120501) has a sorted grain size with a mode of -2.5ϕ (Supplementary Fig. S1). The -4ϕ peak is caused by larger size bombs beyond our sieving range. (Supplementary Fig. S1).

Sukumoyama scoria cone The Sukumoyama scoria cone (790 m in bottom diameter, R. Noguchi, unpublished data) is located in the northern HIMVG field. The rock type of this scoria cone is tholeiitic basalt[4]. This scoria cone consists of lower black scoria layers and an upper yellowish-brown altered scoria layer (Supplementary Fig. S2). The samples were collected from the black scoria layer. The Sukumoyama sample (SK15101201) has a sorted grain size distribution with a mode of -2.5ϕ (Supplementary Fig. S1).

1.2 Miyakejima, Japan

Whole Miyakejima samples in this study were products of the 1983 A.D. fissure eruption event, which started on October 3 and ended on October 4[5]. In this event, the initial eruption started from the southwestern flank of the Oyama volcano with a NE–SW trend, and then fissure vents propagated toward the northeast and southwest[5]. The rock type formed in this event is augite basalt[6].

MJ16102402 and MJ16102403 MJ16102402 and MJ16102403, produced by a typical fire fountain[5], were collected on the eastern rim of the northern Jinanyama scoria cone. At this site, samples were collected on an asphalt-paved ground, which was a ground surface before the 1983 A.D. fissure eruption event (Supplementary Fig. S3). Samples were collected from the lower (MJ16102402) and upper (MJ16102403) parts, respectively (Supplementary Fig. S3). Both samples have sorted grain size distributions with a mode of -3ϕ (Supplementary Fig. S1).

NP15113001–06, and NP16102407 A preatomagmatic eruption in the S vent[5] produced a tuff ring in the Nippana area of southern Miyakejima Island. The edifice originally had a bottom diameter of 400 m and was 25 m in height, and half of it was destroyed by subsequent typhoons and erosions after formation[7]. We collected seven NP samples from the lower to upper layers at the crosscut outcrop, NP15113001 for the lowest layer and NP16102407 for the uppermost layer (Fig. 1, Supplementary Fig. S3). These sample has a poorly sorted grain size distribution (Supplementary Fig. S1). Excluding large counts due to the sieving range, the modes of the grain size distributions are -0.5ϕ for NP1511001 and NP15113002, -1ϕ for NP16102407, -1.5ϕ for NP15113004, -2ϕ for NP15113003 and NP15113005, and -2.5ϕ for NP15113006.

1.3 Myvatn, Iceland

The Myvatn samples investigated in this study were collected from edifices of rootless cones, which are pervasively distributed around the Myvatn region in northern Iceland (Fig. 1). In 2170±38 cal yr BP[8], basaltic lava (*Younger Laxárhraun*[9]) erupted from fissure swarms, and then flowed over wetlands, lakes (including old Lake Myvatn), rivers, and flood plains before reaching the northern bay. Explosive interactions between the lava and wet substrate sediments created more than a hundred rootless cones over the lava surface[10]. The duration of this eruption is estimated to be 30 days based on the analysis of the lava thickness using an equation[11] reported by Höskuldsson et al.[12]. Although typical cones have simple conical edifices with a summit crater (single rootless cones [SRCs]), some rootless cones have smaller cones inside the summit crater (double rootless cones [DRCs])[13]. MY13091004 and MY13091006 were collected from a DRC, and others were collected from SRCs (Figs. 1, Supplementary Fig. S4). There is no layering and grading at outcrops of Myvatn rootless cones that we observed.

MY13091004 and MY13091006 For a DRC on Geitey Island in Lake Myvatn, samples were collected from the outer conical edifice (referred to as the outer cone[13]; MY13091004) and the inner conical edifice (referred to as the inner cone[13]; MY13091006), as shown in Fig. 1. This DRC has bottom diameters of 142 m and 34 m for the outer and inner cones, respectively[13]. MY13091004 is dominated by scoriae and has bombs, while MY13091006 is dominated by spatters. The outer cone and the inner cone have sorted grain size distributions with modes of -2ϕ and -5ϕ, respectively (Supplementary Fig. S1).

MY13091305 and MY13091306 The MY13091305–MY13091306 pair were taken from the same cone (67 m in bottom diameter[13]). MY13091305 and MY13091306 were collected from the bottom and middle of the crosscut outcrop, respectively (Supplementary Fig. S4). MY13091305 has a sorted grain size distribution with a mode of -2ϕ (Supplementary Fig. S1).

MY13091402 MY13091402 was sampled from a rootless cone (51 m in bottom diameter[13]) in Hagi, which is located 45 km from the fissure vents (Fig. 1). Since there was no cross-cut outcrop, we collected the sample from the top of the cone (Supplementary Fig. S4). MY13091402 has a sorted grain size distribution with a mode of -3ϕ (Supplementary Fig. S1).

MY13092002 MY13092002 was collected from the bottom of a large rootless cone (112 m in bottom diameter[13]). This sample is poorly sorted relative to the other Myvatn samples (Supplementary Fig. S1).

2 Microscope observations for the FN, NP, and MY samples

We observed ash particles (2ϕ to 3ϕ) of the Funabara, Nippana, and Myvatn samples using a digital microscope (VHX-2000, KEYENCE) for verification of the CNN–cluster analysis classification result. First, we observed dispersed ash particles. Furthermore, we prepared cross-sectional samples of the ash particles, and then observed them using a magnification of 500× under both incident and reflected light. We measured bubbles in ash particles using the ImageJ software (http://imagej.nih.gov/ij/). The bubbles include those estimated by the arc appearing in the outline of the particle.

The Funabara samples are dominantly red oxidized opaque grains (Fig. 1 and Supplementary Fig. S5), and the surfaces of the particles appear glassy under incident lighting. At 500× magnification, the samples contain microlites (plagioclase, olivine, and magnetite). Some of the particles are coated with red oxidized magna and are therefore opaque in transmission mode (e.g., in FN15101208, Supplementary Fig. S5). Transparent particles in the Funabara samples are brownish-yellow grains and freecrystals (plagioclase, olivine, and pyroxene). Brownish-yellow grains, which have a glassy surface, are more dominant in FN15101206.

The bubble size of the Funabara ash particles is between 16 and 107 μm.

Most of the particles in the Nippana samples are glass fragments, which often contain microlites (plagioclase and magnetite) on the microscopic scale (Fig. 1 and Supplementary Fig. S6). The other particles are free from crystals (plagioclase). NP16102407 contains black opaque grains, which are highly microcrystalline. Qualitatively, the characteristics of the grain shapes differ among samples, having concave shapes (NP15113001, NP15113002, and NP15113003) and rectilinear edges (NP15113004, NP15113005, and NP15113006). The bubble size of the Nippana samples is between 9 and 181 μm.

Most of the particles in the Myvatn samples are glassy (Fig. 1 and Supplementary Fig. S7). Plagioclases are found as both free crystals and microlites. Some particles have patchy textures due to heterogeneous crystallization (e.g., in MY13091306, Supplementary Fig. S7). In the transmission-mode observations, transparent grains are dominant in MY13091004 and MY13091305, and opaque grains are dominant in MY13091306 and MY13091402, qualitatively. MY13091402 includes hollow spherules (Supplementary Figs. S7, S8). The bubble size of Myvatn samples is between 13 and 300 μm.

3 Transparency of samples

Using a Morphologi G3S^{TM}, we obtained the following transparency parameters for each image[14], the mean (I_m) and standard deviation (I_{SD}) of the intensity:

$$I_m = \frac{\sum_{i=1}^{N} I_i}{N} \tag{1}$$

$$I_{SD} = \sqrt{\frac{\sum_{i=1}^{N} {I_i}^2 - \left(\sum_{i=1}^{N} I_i\right)^2 / N}{N}} \tag{2}$$

where I_i is the intensity (0–255) of the ith pixel, and N is the total number of pixels for one grain. I_m and I_{SD} were calculated for every grain. For the cluster analysis of the samples, in order to obtain the overall transparency values for each sample, we calculated the mean,

median, and standard deviation of I_m and I_{SD}.

4 Particle shape parameters in Table 3

In Table 3 shows values of general particle shape parameters to demonstrate our quantitative particle selection. We chose four parameters: aspect ratio (A_r), convexity (C_v), HS (high sensitivity) circularity (H_c), and solidity (S_d). These parameters are derived as follows:

$$A_r = \frac{W}{L} \tag{3}$$

$$C_v = \frac{P_c}{P_g} \tag{4}$$

$$H_c = \frac{4 \times \pi \times A_g}{{P_g}^2} \tag{5}$$

$$S_d = \frac{A_g}{A_g + A_c} \tag{6}$$

where W is the length along the minor axis of the grain, L is the length along the major axis of the grain, P_c is the perimeter of the convex hull, P_g is the perimeter of the grain, A_c is the area of the convex hull, and A_g is the area of the grain[14]. We obtained these parameter values using the ImageJ software (http://imagej.nih.gov/ij/). The threshold for the binarization is 0–128. After the four parameters for every basal image were calculated, we averaged them over each basal shape.

References

1. Aramaki, S. & Hamuro, K. Geology of the Higashi-Izu Monogenetic Volcano Group. *Bull. the Earthquake Res. Inst., Univ. Tokyo,* **52**(2), 235–278 (1977).

2. Yusa, Y. & Kuroda, N. Geology and petrology of Izu-Takatsukayama and Funabara volcano. *Geoscience reports of Shizuoka Univ.,* **2**(1), 43–54 (1970) (In Japanese).*

3. Suzuki, Y. Petrogenesis of felsic magma in Higashi–lzu monogenetic volcano group. *Bull. Volcanol. Soc. Japan,* **45**, 149–171 (2000) (in Japanese with English abstract).

4. Kikuchi, K. & Takahashi, M. Petrology for volcanic rocks produced by nearly contemporaneous eruptions of aligned volcanic centers in the Higashi-Izu Monogenetic Volcano Group, central Japan. *Bull. Inst. Natural Sci., College of Humanities and Sci., Nihon Univ.,* **39**, 217–246 (2004) (in Japanese with English abstract).

5. Aramaki, S., Hayakawa, Y., Fujii, T., Nakamura, K. & Fukuoka, T. The October 1983 eruption of Miyakejima volcano, *J. Volcanol. Geotherm. Res.,* **29**(1–4), 203–229 (1986).

6. Fujii, T., Aramaki, S., Fukuoka, T. & Chiba, T. Petrology of the ejecta and lavas of the 1983 eruption of Miyake-jima. *Bull. Volc. Society of Japan*, **2**, (29), 266–282 (1984) (in Japanese with English abstract).

7. Sumita, M. Ring-shaped cone formed during the 1983 Miyake-jima eruption. *Bull. Volcanol. Soc. Japan,* **30**, 11–32 (1985) (in Japanese with English abstract).

8. Hauptfleisch, U. High-resolution palaeolimnology of Lake Mývatn, Iceland. *PhD thesis of University of Iceland* , Reykjavik (2012).

9. Thorarinsson, S. The crater groups in Iceland. *Bull. volcanologique,* **2**, 1–44 (1953).

10. Einarsson, Á. The palaeolimnology of Lake Mývatn, northern Iceland: plant and animal microfossils in the sediment. *Freshwater Biology,* **12**, 63–82 (1982).

11. Hon, K., Kauahikaua, L., Denlinger, R. & Mackay, K. Emplacement and inflation of pahoehoe sheet flows: observations and measurements of active lava flows on Kilauea Volcano, Hawaii. *Geol. Society of America Bull.,* **106**, (3), 351–370 (1994).

12. Höskuldsson, Á., Dyhr, C. & Dolvik, T. Grænavatnsbruni og Laxárhraun yngra. *Haustrá dstefna Jardfrædafélags Íslands, Ágrip erinda,* 41–44 (2010) (in Icelandic).

13. Noguchi, R., Höskuldsson, Á. & Kurita, K. Detailed topographical, distributional, and material analyses of rootless cones in Myvatn, Iceland. *J. Volcanol. Geotherm. Res.,* **318**, 89–102 (2016).

14. Malvern Instruments Ltd. Morphologi G3 User Manual. 280 p (2013).

*Title etc. translated by R.N.

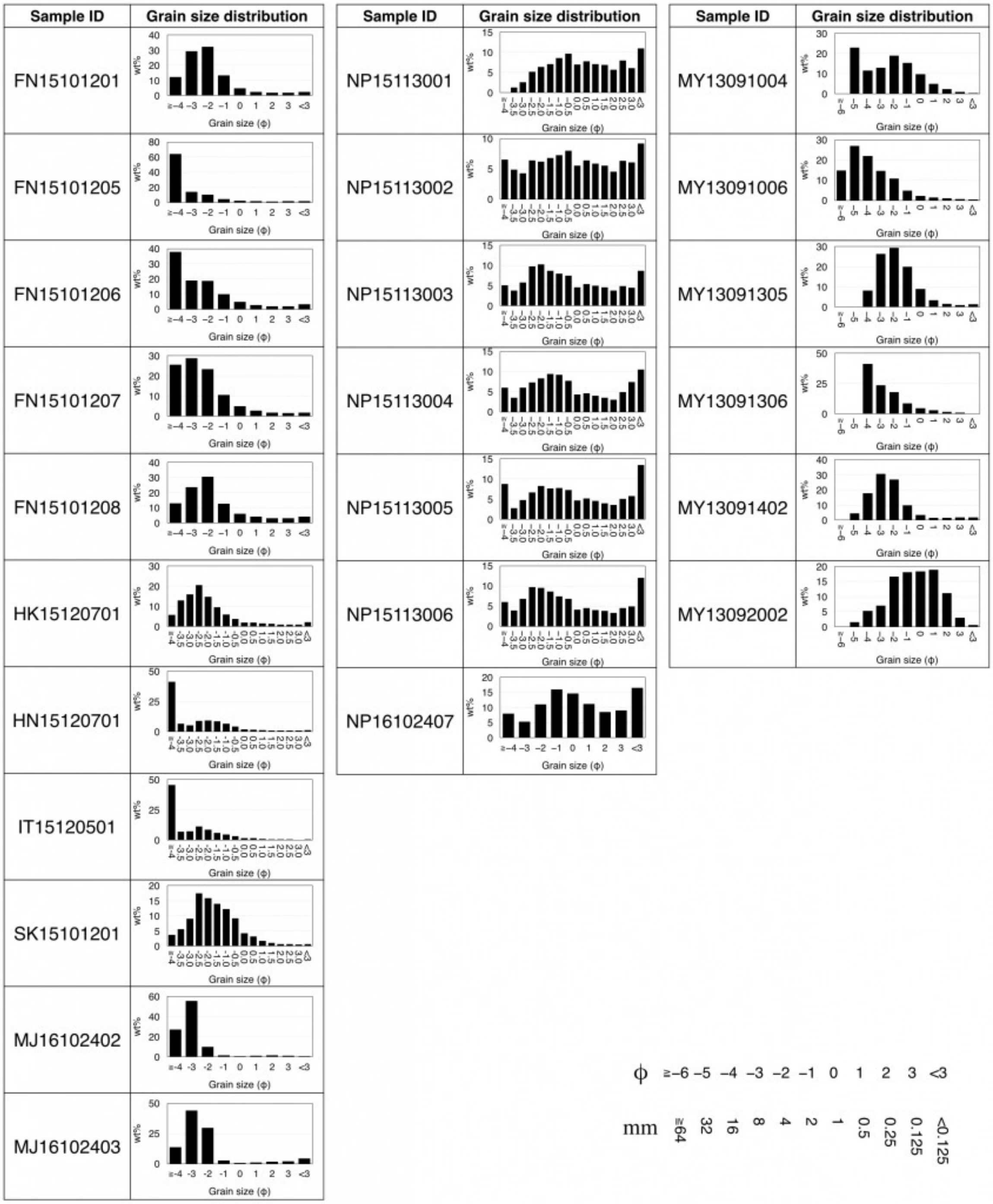

Figure S1: Grain size distribution for each sample in this study.

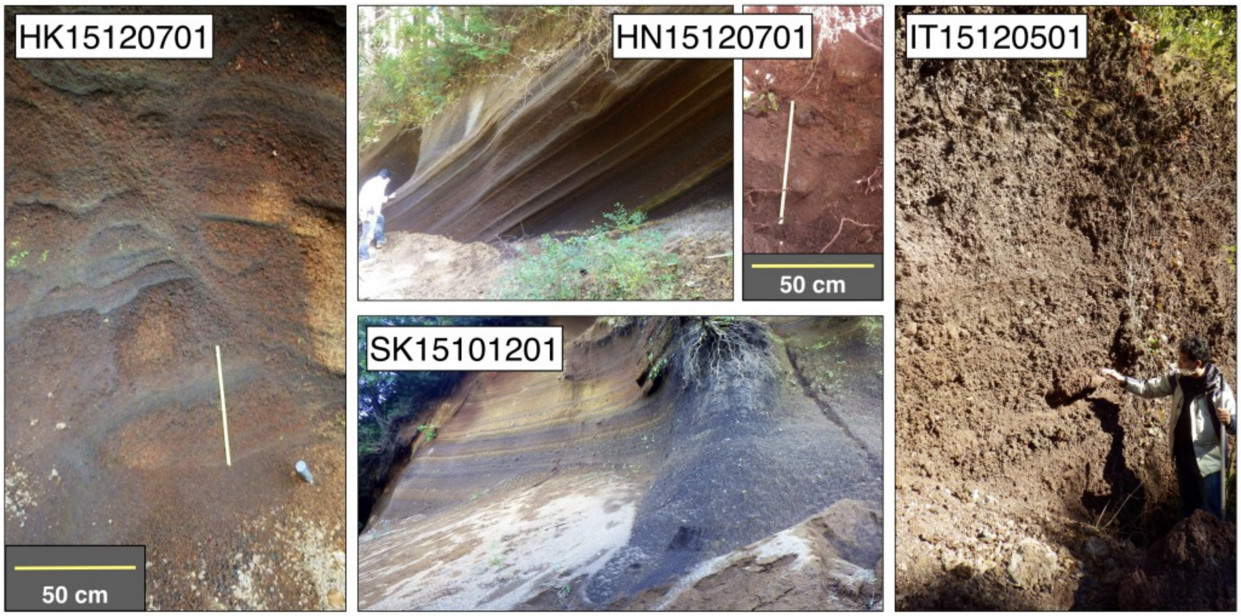

Figure S2: Outcrops, which were samples collected from Izu Peninsula, Japan.

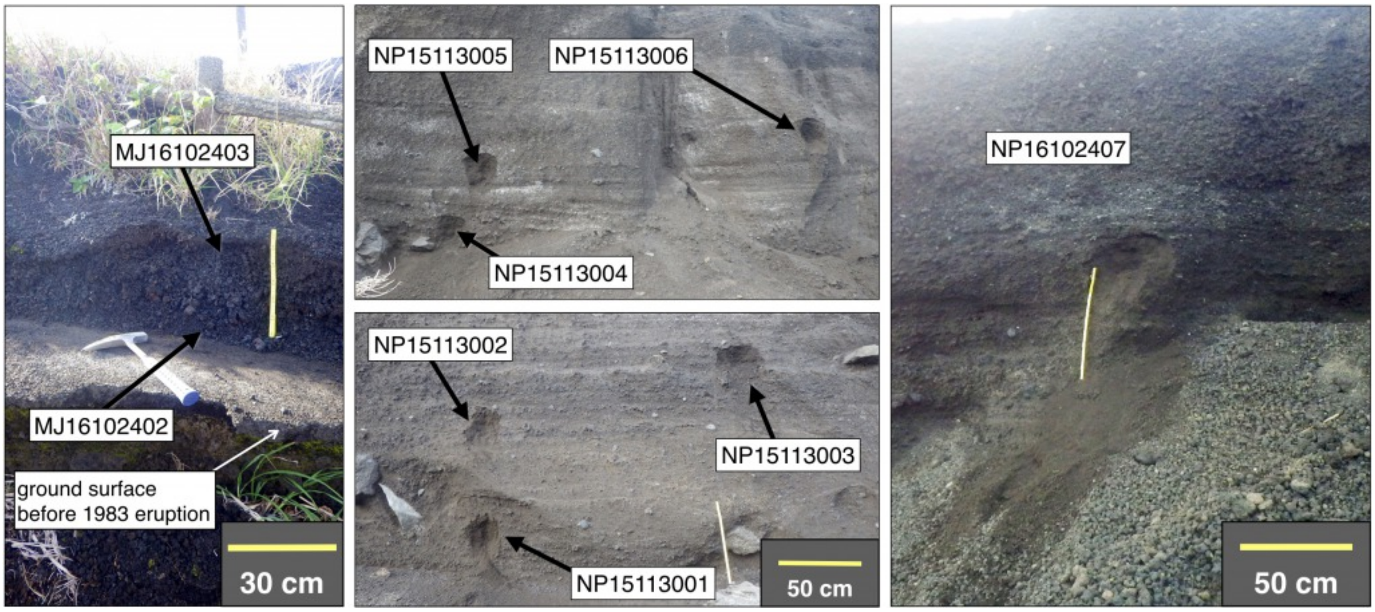

Figure S3: Outcrops, which were samples collected from Miyakejima Island, Japan.

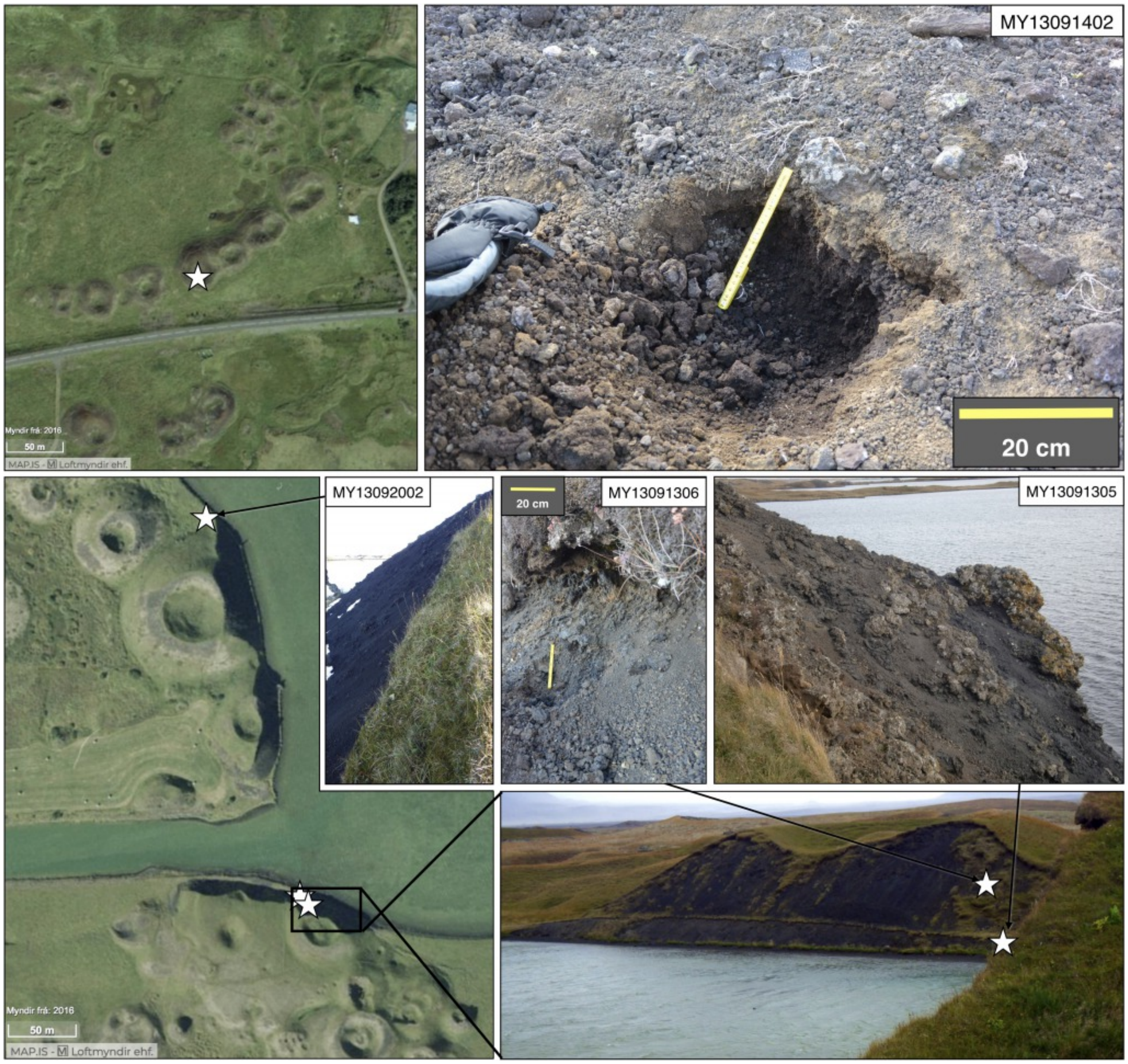

Figure S4: Outcrops, which were samples collected around Myvatn, Iceland. Basal aerial photos were taken by Loftmyndir ehf.

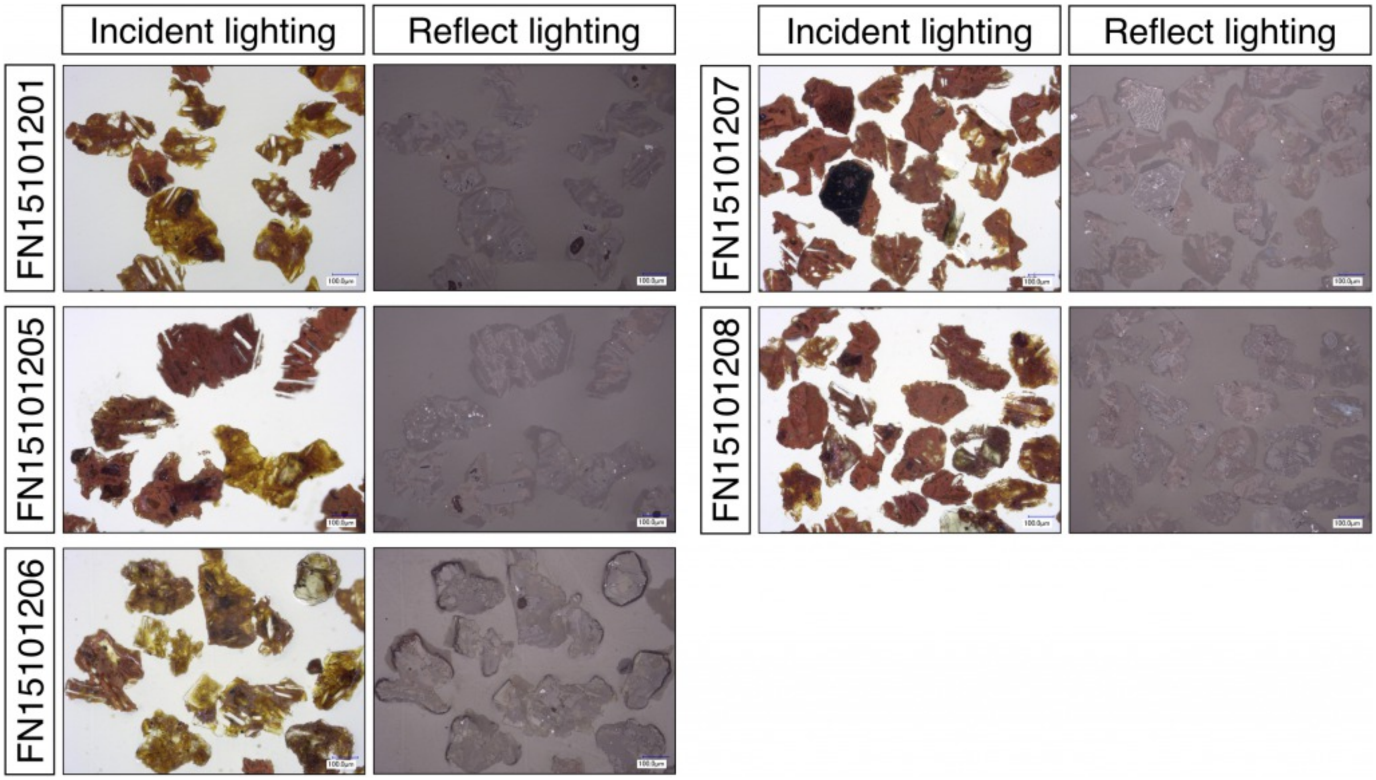

Figure S5: Cross-sectional images of each Funabara sample.

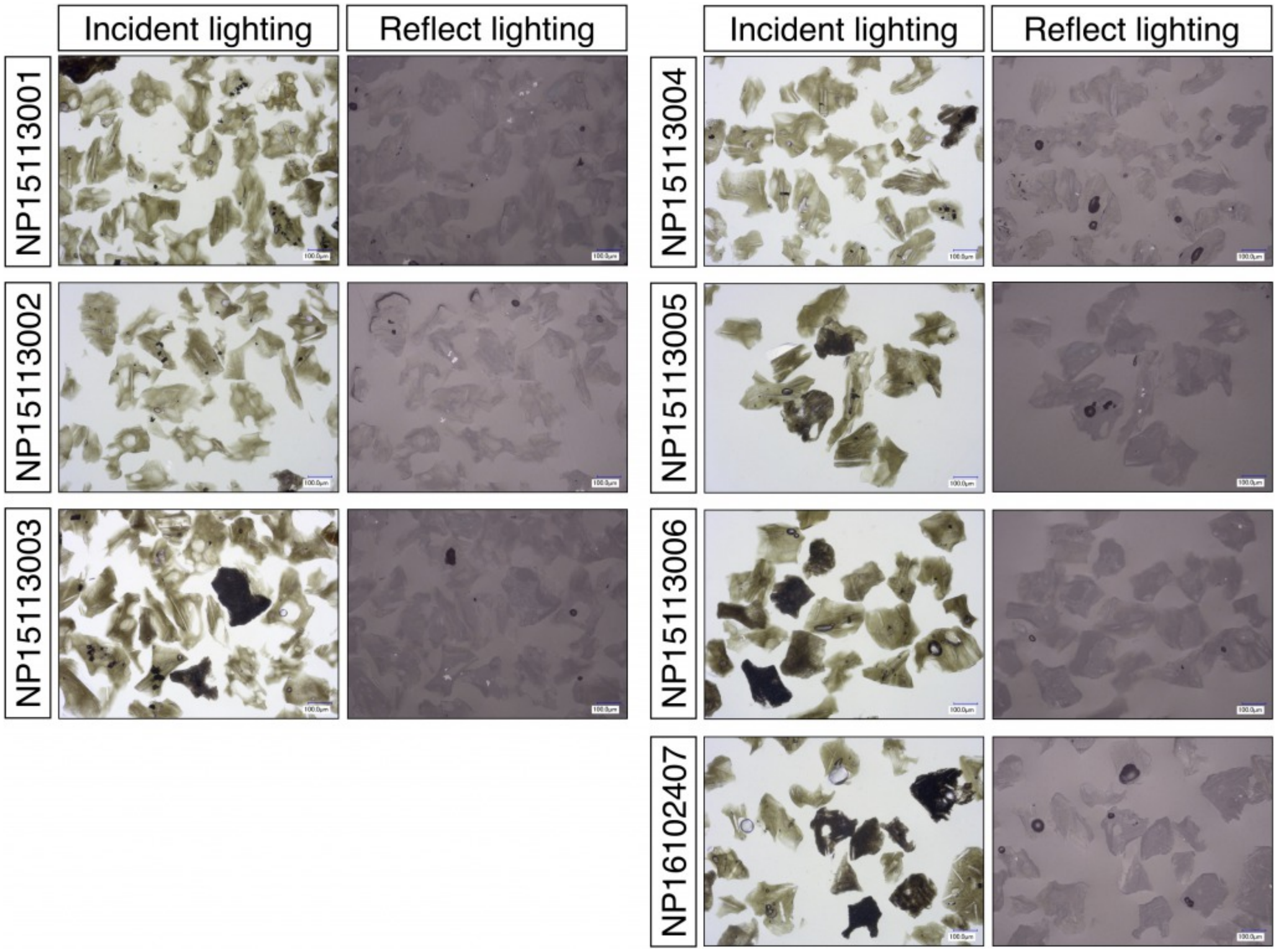

Figure S6: Cross-sectional images of each Nippana sample.

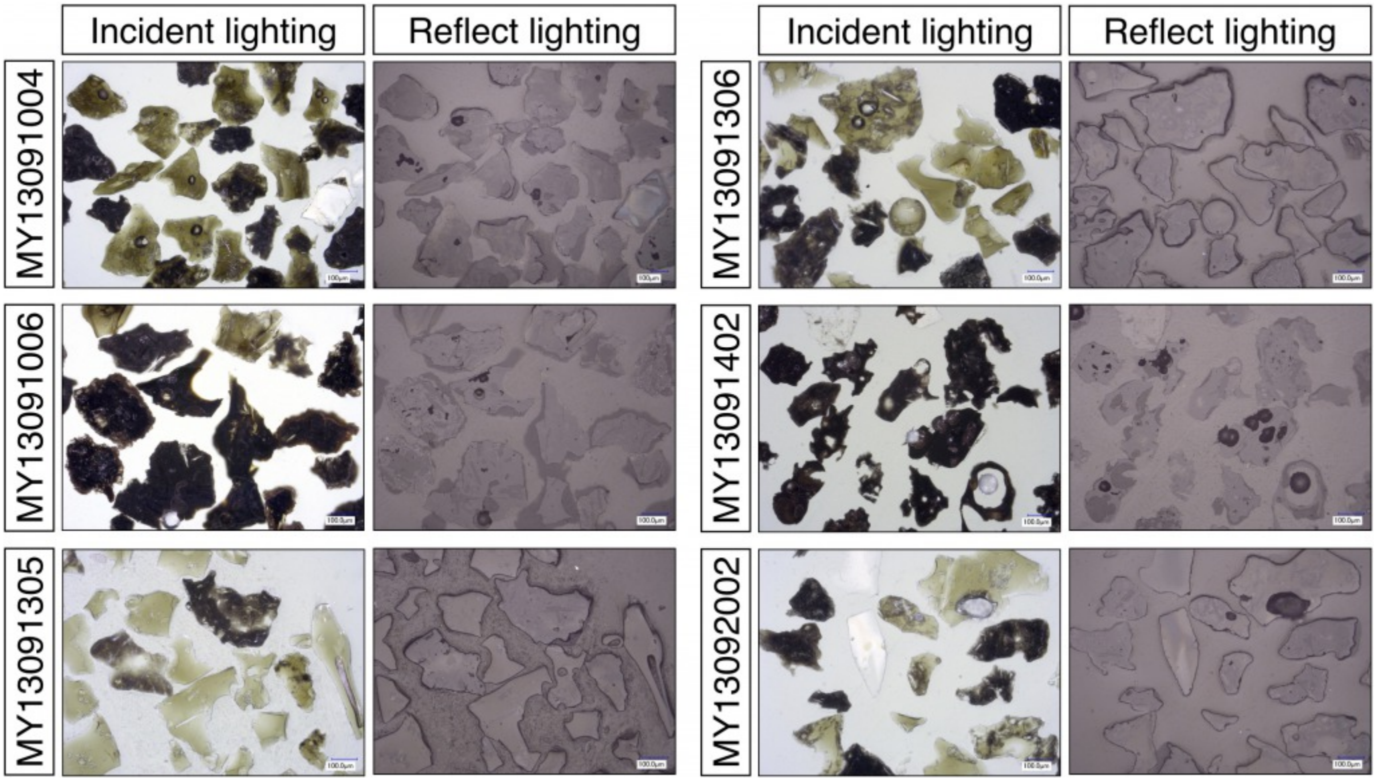

Figure S7: Cross-sectional images of each Myvatn sample.

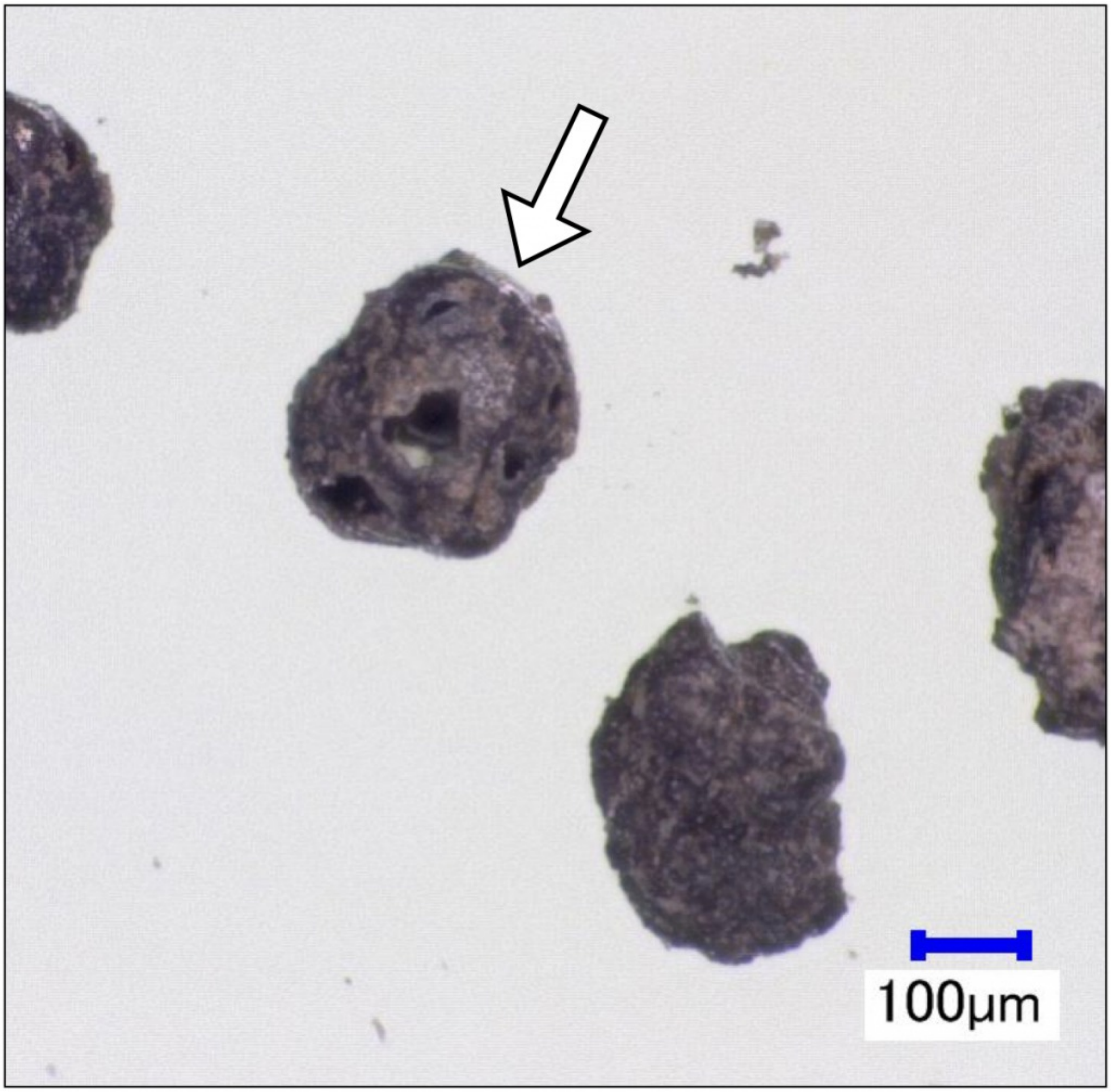

Figure S8: Example of hollow spherule (white arrow) in MY13091402.
